# Immune-mediated inflammatory diseases differently affect IGRAs’ accuracy for latent tuberculosis infection diagnosis in clinical practice

**DOI:** 10.1371/journal.pone.0189202

**Published:** 2017-12-07

**Authors:** Irene Latorre, Sonia Mínguez, José-Manuel Carrascosa, Juan Naves, Raquel Villar-Hernández, Beatriz Muriel, Cristina Prat, Esther García-García, Irma Casas, Eugeni Domènech, Carlos Ferrándiz, Lourdes Mateo, Jose Domínguez

**Affiliations:** 1 Servei de Microbiologia, Hospital Universitari Germans Trias i Pujol, Institut d’Investigació Germans Trias i Pujol, Badalona, Spain; 2 Universitat Autònoma de Barcelona, Bellaterra, Barcelona, Spain; 3 CIBER Enfermedades Respiratorias, Instituto de Salud Carlos III, Badalona, Spain; 4 Servei de Reumatologia, Hospital Universitari Germans Trias i Pujol, Institut d’Investigació Germans Trias i Pujol, Badalona, Spain; 5 Servei de Dermatologia, Hospital Universitari Germans Trias i Pujol, Institut d’Investigació Germans Trias i Pujol, Badalona, Spain; 6 Servei de Digestiu, Hospital Universitari Germans Trias i Pujol, Institut d’Investigació Germans Trias i Pujol, Badalona, Spain; 7 Servei de Medicina Preventiva, Hospital Universitari Germans Trias i Pujol, Institut d’Investigació Germans Trias i Pujol, Badalona, Spain; 8 CIBER Enfermedades Hepáticas y Digestivas, Instituto de Salud Carlos III, Badalona, Spain; University of Cape Town, SOUTH AFRICA

## Abstract

**Background:**

Clinical accuracy of IGRAs remains unclear on patients with immune-mediated inflammatory diseases (IMIDs). Here, we assess the impact of immunosuppressants and IMIDs on QuantiFERON-TB Gold In-Tube (QFN-G-IT) and T-SPOT.TB accuracy.

**Methods:**

Patients with IMIDs who required latent tuberculosis infection (LTBI) screening were enrolled and classified into: (i) 50 patients with inflammatory rheumatic diseases, (ii) 50 patients with psoriasis and (iii) 30 patients with Crohn’s disease. A total of 44 healthy individuals without immunosuppression were also included as controls. Tuberculin skin test (TST), T-SPOT.TB and QFN-G-IT assays were performed. IGRAs were performed following manufacturer’s instructions.

**Results:**

Immunosuppressant’s intake was more frequent on patients with Crohn’s disease and psoriasis. Positive IGRAs and TST results were reduced in Crohn’s disease patients, whereas rate of indeterminate T-SPOT.TB results was increased in this group with respect to the other IMIDs analysed and controls. When IFN-γ response was studied, the levels of this cytokine after mitogen stimulation were significantly lower in Crohn’s and inflammatory rheumatic diseases than in psoriasis. Interestingly, psoriatic patients were the only ones not receiving corticosteroids. Furthermore, a negative correlation was observed between the IFN-γ secreted after mitogen stimulation and corticosteroids dose.

**Conclusions:**

IMIDs seem to negatively affect the clinical accuracy of IGRAs, being Crohn’s disease patients the most affected individuals due to their concomitant drug-profile and impaired immune response.

## Introduction

Patients with immune-mediated inflammatory diseases (IMIDs) have a higher risk of tuberculosis (TB) reactivation than common population because of the intake of different immunosuppressive therapies and/or their main disease itself. Tumour Necrosis Factor (TNF)-alpha (α) is a pro-inflammatory key cytokine playing an important role in the immune response against *Mycobacterium tuberculosis*. It is crucial for maintaining the granuloma formation and thus containing the infection caused by the bacilli. Therefore, ruling out latent tuberculosis infection (LTBI) and active TB is mandatory prior anti-TNF-αprescription as well as during treatment [1, 2].

Interferon (IFN)-gamma (γ) Release Assays (IGRAs) used for LTBI diagnosis detect the IFN-γ cytokine secreted by sensitized T-cells after stimulating with specific *M*. *tuberculosis* antigens [3]. There are two assay formats: QuantiFERON technology (QFN; Qiagen, Düsseldorf, Germany) and T-SPOT.TB (Oxford Immunotec, Abingdon, UK), both approved by the U.S. Food and Drug Administration (FDA) and the CE (for their use in Europe). Currently, these assays are useful tools for diagnosing LTBI because they are not affected by BCG-vaccination and/or non-tuberculous mycobacteria (NTM) sensitization [4–6]. In addition, IGRAs’ methodology contain a positive control [stimulation with phytohaemagglutinin (PHA) as a mitogen] which detects the presence of anergy. This control is very useful in individuals with a weak immune response due to the intake of several immunosuppressant therapies or the presence of a certain underlying disease. Therefore, the inclusion of this mitogen could be particularly useful in patients with IMIDs [7]. However, clinical performance of IGRAs remains still unclear in these kind of patients. Currently, available data on the usefulness of IGRAs in patients with IMIDs is diverse due to the population heterogeneity and the variety of immunosuppressant drug-regimens analysed [8, 9]. Therefore, research in this direction should be further considered. In the present study, we have investigated the impact of different immunosuppressant therapies and IMIDs on QFN-TB Gold In-Tube (QFN-G-IT) and T-SPOT.TB assays.

## Materials and methods

### Study participants

A total of 130 patients and 44 healthy controls were enrolled in the study. Samples were collected from Rheumatology, Dermatology, Gastroenterology and Preventive Medicine Departments located in Hospital Universitari Germans Trias I Pujol (Badalona, Spain). The study was approved by the Ethics Committee of the Hospital Germans Trias i Pujol. All experiments were performed in accordance with the relevant guidelines and regulations. All the patients enrolled in this study gave a written informed consent. A total of 11 mL of blood was collected from each patient.

### Tuberculin skin testing

Tuberculin skin test (TST) was administered using the Mantoux method and interpreted according to Spanish Society of Pneumology Guidelines by qualified members of the research team. TST was considered positive when the induration was higher than 5mm [10]. In order to avoid TST booster effect over IGRAs, TST and blood extraction for T-SPOT.TB and QFN-G-IT were performed simultaneously [11]. The study was double-blinded: TST was interpreted without knowing IGRAs’ results, and at the same time, researchers did not know the clinical data and the TST result prior to performing the tests.

### IFN-γ release assays and results interpretation

T-SPOT.TB and QFN-G-IT assays were performed and interpreted following manufacturer’s instructions. For T-SPOT.TB assay a total of 8 mL of blood were collected. Spot-forming cells (SFC) were counted using an automated AID ELISPOT reader (AID Systems, Strasberg, Germany). The results were also checked and validated by naked eye. The assay was considered positive when the test wells contained at least six SFC more than the negative control, and this number was at least twice the number of the negative control well. The result was considered indeterminate if both antigen-stimulated wells were negative and if the number of SFC in the positive control was less than <20; and/or if SFC counted in the negative control well was>10. For QFN-G-IT a total of 3 mL of blood was collected into three tubes (negative, TB-Antigen and positive). For this assay, a result was considered positive when the IU/mL of IFN-γ secreted was ≥0.35 for the TB-Antigen tube. A result was scored as indeterminate when the TB-antigen tube was negative and the mitogen tube presented less than 0.5 IU/mL of IFN-γ secreted (positive control); and/or if the negative control tube was higher than 8.0 UI/mL. Measurements >10IU/mL in QFN-G-IT were set as 10IU/mL according to the upper limit of the assay and manufacturer’s instructions.

### Statistical analysis

Differences on the assay results based on the clinical therapeutic profile and the underlying disease was assessed and analysed using the Chi-square test. Association between the IFN-γ response and corticosteroid doses was assessed using Spearman correlation coefficient. The amount of IFN-γ secreted was investigated as well in each group. In this case, comparisons among the three study groups were performed using the Kruskall-Wallis test. In addition, Mann-Whitney U test was applied for pairwise comparisons. Differences were considered statistical significant when a p-value was <0.05. All analyses were performed using the SPSS statistical software for windows (SPSS version 15.0; SPSS Inc, Chicago, IL, USA). Graphical representation is based on GraphPad Prism version 4 (GraphPad Software, Inc, San Diego, CA).

## Results

### Patient characteristics and concomitant drug-profile when IGRAs where performed

We enrolled a total of 130 IMID patients who required LTBI screening before starting systemic immunosuppressive treatment or during its sustained use. Patients were screened in the course of their routine examinations and had no known risk of TB exposure. They were classified into three groups: (i) 50 patients with inflammatory rheumatic diseases, (ii) 50 patients with moderate-to-severe psoriasis and (iii) 30 patients with Crohn’s disease. A total of 44 healthy individuals without immunosuppression were also included as controls. They were healthcare workers with no risk of TB exposure and recruited in the course of routine examinations.

Patient characteristics and their specific underlying disease regarding the study group is detailed in Table 1. Briefly, the proportion of patients on immunosuppressants (DMARDs or biologics) when IGRAs were performed varied significantly regarding the underlying disease (p = 0.016). It was higher in patients with Crohn’s disease (90%; 27/30) and psoriasis (82%; 41/50) with respect to inflammatory rheumatic patients (64%; 32/50). The 75% (24/32) of rheumatic patients were receiving one DMARD and the 25% (8/32) were taking two. The 46.3% (19/41) of patients with psoriasis were receiving classic systemic treatment (cyclosporine or methotrexate) and the 53.7% (22/41) were already taking biologicals (infliximab, etanercept, adalimumab or ustekinumab). The 81.5% (22/27), 11.1% (3/27) and 7.4% (2/27) of Crohn’s disease patients were treated with azathioprine, methotrexate and biologicals (infliximab) respectively. Corticosteroid therapy was frequent in patients with inflammatory rheumatic diseases (40%; 20/50) and Crohn’s disease (33.3%; 10/30), while it was absent in psoriatic patients (Table 1).

**Table 1:** Patients’ characteristics, final diagnosis and present treatment regarding IMID type.

VARIABLE	Psoriasis n = 50 (%)	Inflammatory rheumatic diseases n = 50 (%)	Crohn’s disease n = 30 (%)	Healthy controls n = 44 (%)
**Mean age (years) ± SD**	45.52±13.05	49.58±13.11	38.16±13.94	36.21±7.22
**Gender**				
Male	37 (74)	17 (34)	16 (53.3)	10 (22.7)
Female	13 (26)	33 (66)	14 (46.7)	34 (77.3)
**Diagnosis**				
Moderate-to-severe psoriasis	50 (100)	0 (0)	0 (0)	N/A
Rheumatoid arthritis	0 (0)	16 (32)	0 (0)	N/A
Ankylosing spondylitis	0 (0)	12 (24)	0 (0)	N/A
Psoriatic arthritis	0 (0)	9 (18)	0 (0)	N/A
Crohn’s disease	0 (0)	0 (0)	30 (100)	N/A
Other*	0 (0)	13 (26)	0 (0)	N/A
**Present corticoids**				
Yes	0 (0)	20 (40)	10 (33.3)	N/A
No	50 (100)	30 (60)	20 (66.7)	N/A
**Present DMARDs**				
Yes	19 (38)	32 (64)	25 (83.3)	N/A
No	31 (62)	18 (36)	5 (16.7)	N/A
**Present biologics**				
Yes	22 (44)	0 (0)	2 (6.7)	N/A
No	28 (56)	50 (100)	28 (93.3)	N/A

SD: standard deviation

*Systemic lupus erythematosus (n = 4), SAPHO syndrome (n = 4), seronegativepolyarthritis (n = 3), undifferenciated spondiloarthropaty (n = 1) and spondylitis associated with inflammatory bowel disease (n = 1).

### IGRAs’ clinical accuracy regarding IMID type

**Fig 1:**
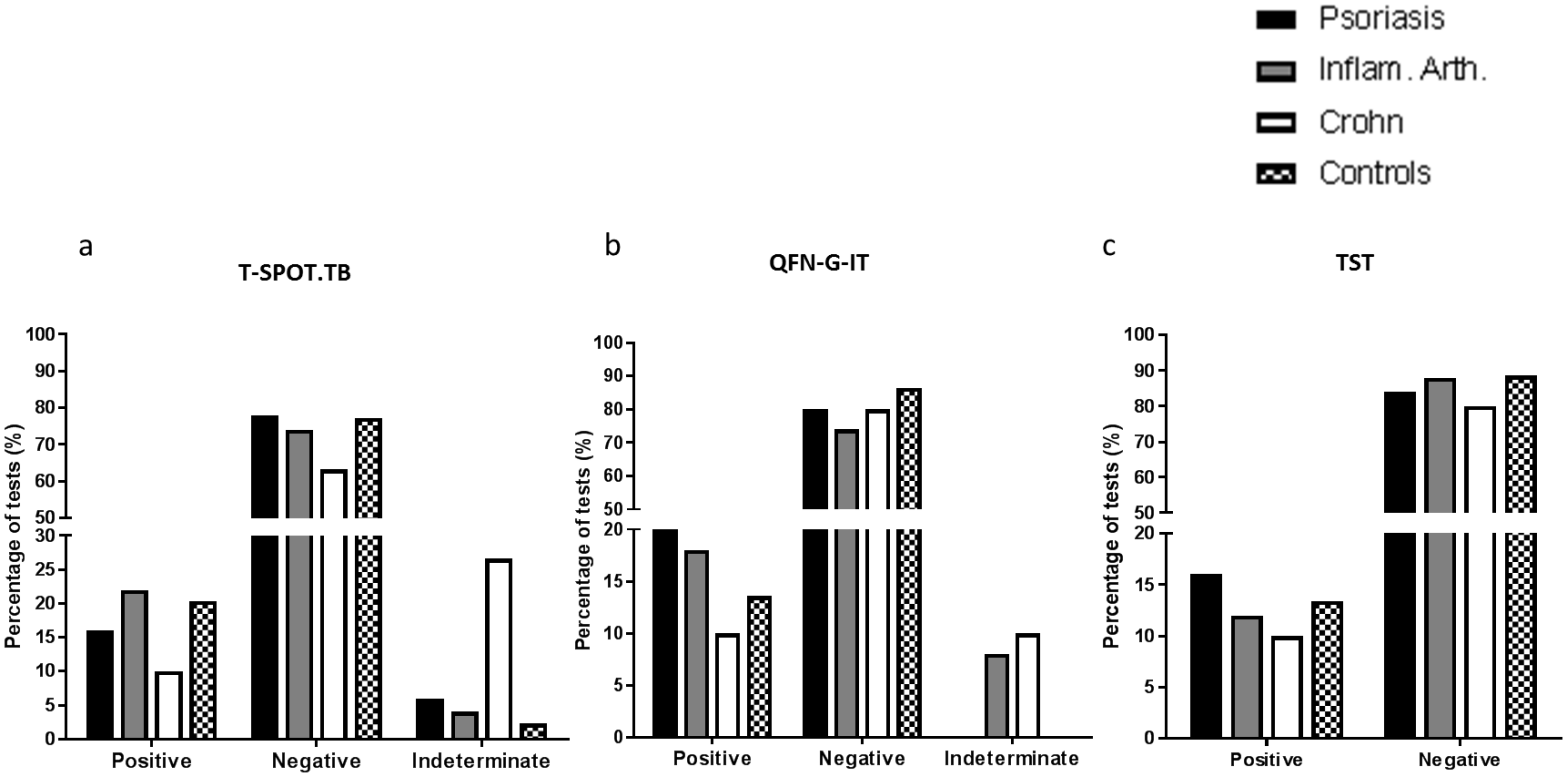
Percentage of positive, negative and indeterminate results. Percentages are represented for (a) T-SPOT.TB, (b) QFN-G-IT and (c) TST in patients with psoriasis (black bars), inflammatory arthritis diseases (grey bars), Crohn disease (white bars) and healthy controls (spot bars). Inflam. Arth: Inflammatory arthritis; QFN-G-IT: QuantiFERON-TB Gold In-Tube; TST: Tuberculin skin test.

Overall, the percentage of positive results by either of the two IGRAs were 22.7% (10/44), 22% (11/50), 24% (12/50), and 13.3% (4/30) in healthy controls, patients with psoriasis, rheumatic inflammatory diseases and Crohn’s disease respectively. In addition, a considerable percentage of IMIDs patients with negative TST (12.7%; 14/110) were positive for one of the two IGRAs. The percentage of positive results in patients with psoriasis, inflammatory arthritis, Crohn’s disease and healthy controls is represented for the three assays in Fig 1A–1C. The rate of positive results obtained by T-SPOT.TB, QFN-G-IT and TST was higher in patients with inflammatory rheumatic diseases, psoriasis and healthy controls with respect to Crohn’s disease individuals (differences not significant). Indeterminate results were obtained by T-SPOT.TB and QFN-G-IT in a 10% [13/130; 10 cases due to insufficient mitogen (PHA) response and 3 due to high response in negative control) and a 5.4% (7/130; all due to insufficient PHA response)] of the cases respectively. All indeterminate results due to insufficient PHA response corresponded with negative TSTs. The percentage of indeterminate results observed by T-SPOT.TB in patients with Crohn’s disease diagnosis was significantly higher than in rheumatic patients, those diagnosed with psoriasis and healthy controls (p = 0.003, p = 0.009 and p = 0.002 respectively). Seven out of the eight Crohn’s disease patients with indeterminate results corresponded to individuals receiving azathioprine. Furthermore, three of them were receiving corticosteroid therapy. Concordances between both IGRAs were good when they were analysed globally (κ = 0.817) and based on the study groups (κ = 0.849 for rheumatic inflammatory diseases, κ = 0.799 for psoriasis and κ = 0.773 for Crohn’s disease). By means of an univariate analysis, no significant differences were observed between the results obtained by either IGRAs or TST with biologics, corticoids or DMARDs received (Table 2).

**Table 2:** Association of the immunosuppressant treatment received with TST, QFN-G-IT and T-SPOT.TB positivity by means of univariate analysis.

Risk Factors	TST; n (%)			QFN-G-IT; n (%)			T-SPOT.TB; n (%)		
	Positive	Negative	p-value	Positive	Negative	p-value	Positive	Negative	p-value
**Total**^a^	17	110	NA	22	101	NA	22	95	NA
**Mean age (years) ± SD**^b^	52.53±12.50	44.42±14.47	0.031	57.19±10.68	43.62±13.81	<0.0001	56.76±13.16	43.71±12.64	<0.0001
**Gender**									
Male	11 (64.7)	56 (50.9)	0.289	18 (81.8)	51 (50.5)	0.007	17 (77.3)	46 (48.4)	0.014
Female	6 (35.3)	54 (49.1)		4 (18.2)	50 (49.5)		5 (22.7)	49 (51.6)	
**Present immunosuppressants (DMARDs or biologics)**									
Yes	12 (70.6)	85 (77.3)	0.548^c^	17 (77.3)	78 (77.2)	0.996	15 (68.2)	76 (80.0)	0.259^c^
No	5 (29.4)	25 (22.7)		5 (22.7)	23 (22.8)		7 (31.8)	19 (20.0)	
**Present DMARDs**									
Yes	9 (52.9)	66 (60.0)	0.582	11 (50.0)	60 (59.4)	0.418	11 (50.0)	58 (61.1)	0.342
No	8 (47.1)	44 (40.0)		11 (50.0)	41 (40.6)		11 (50.0)	37 (38.9)	
**Present biologics**									
Yes	3 (17.6)	19 (17.3)	1.000^c^	6 (27.3)	18 (17.8)	0.373^c^	4 (18.2)	18 (18.9)	1.000^c^
No	14 (82.4)	91 (82.7)		16 (72.7)	83 (82.2)		18 (81.8)	77 (81.1)	
**Present corticoids**									
Yes	4 (23.5)	26(23.6)	1.000^c^	5 (22.7)	22(21.8)	0.923	5 (22.7)	22(23.2)	0.966
No	13 (76.5)	84(76.4)		17 (77.3)	79(78.2)		17 (77.3)	73(76.8)	

NA: non-applicable; SD: standard deviation. P vàlues less than 0.05 were considered significant.

^a^Excluding indeterminate QFN-G-IT and/or T-SPOT.TB results.

^b^Significance on age was calculated using Student’s T test.

^c^Significance was calculated using Fisher’s Exact test, since the 25% of expected frequency were less than 5.

### Impact of immunosuppressive therapies on the IFN-γ response

**Fig 2:**
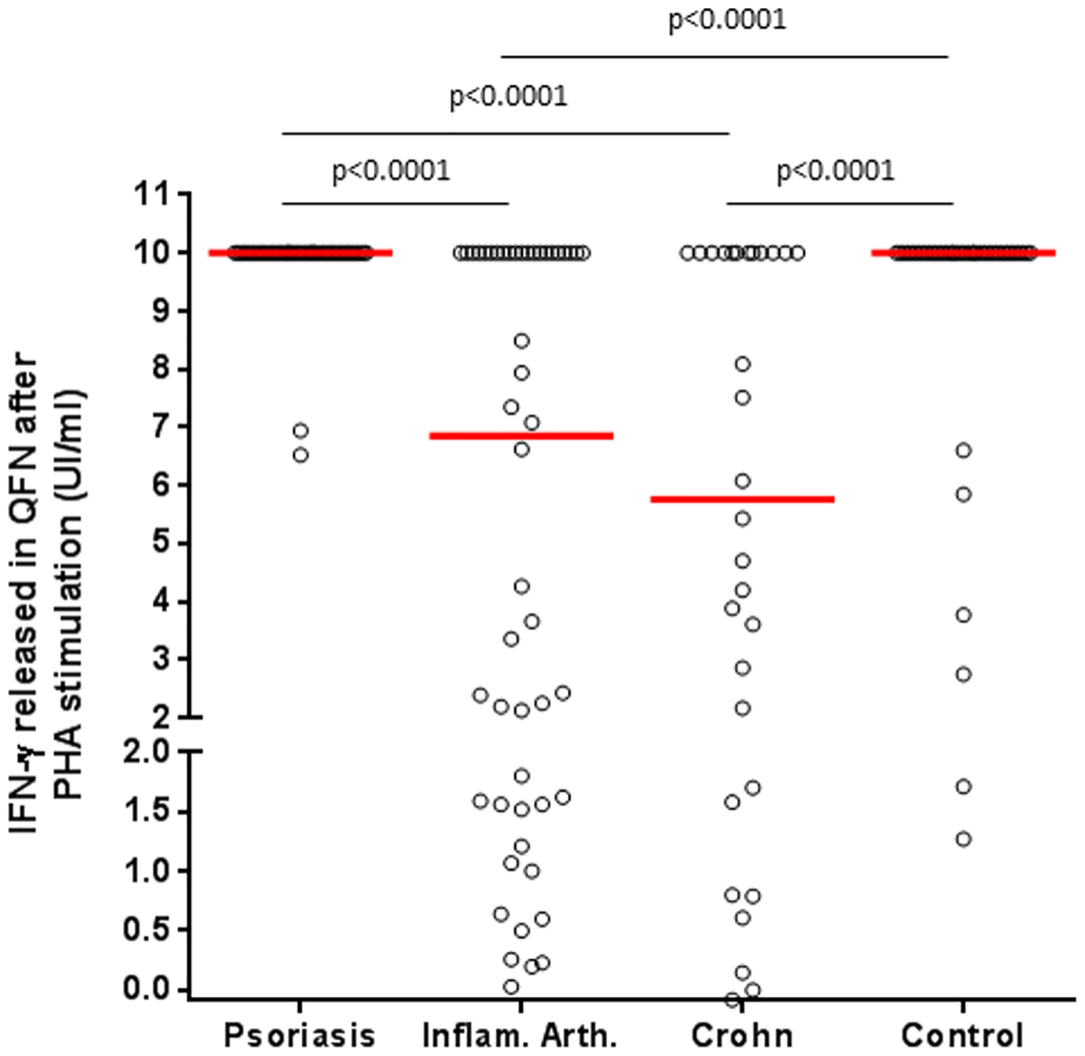
IFN-γ released after mitogen (PHA) overnight stimulation (Mit-Nil) in QFN-G-IT based on the study group of patients analyzed. The median cytokine levels from each group is represented by a line. Mann-Whitney U analysis was applied for pairwise comparisons. Measurements >10IU/mL were set as 10IU/mL according to the upper limit of the assay and manufacturer’s instructions. Only significant differences were indicated in the figure. It was not possible to assess the IFN-γ response after PHA stimulation in T-SPOT.TB assay due to saturation in the control well (>250 spot-forming cells). Inflam. Arth: Inflammatory arthritis; PHA: Phytohaemagglutinin; QFN-G-IT: QuantiFERON-TB Gold In-Tube.

**Fig 3:**
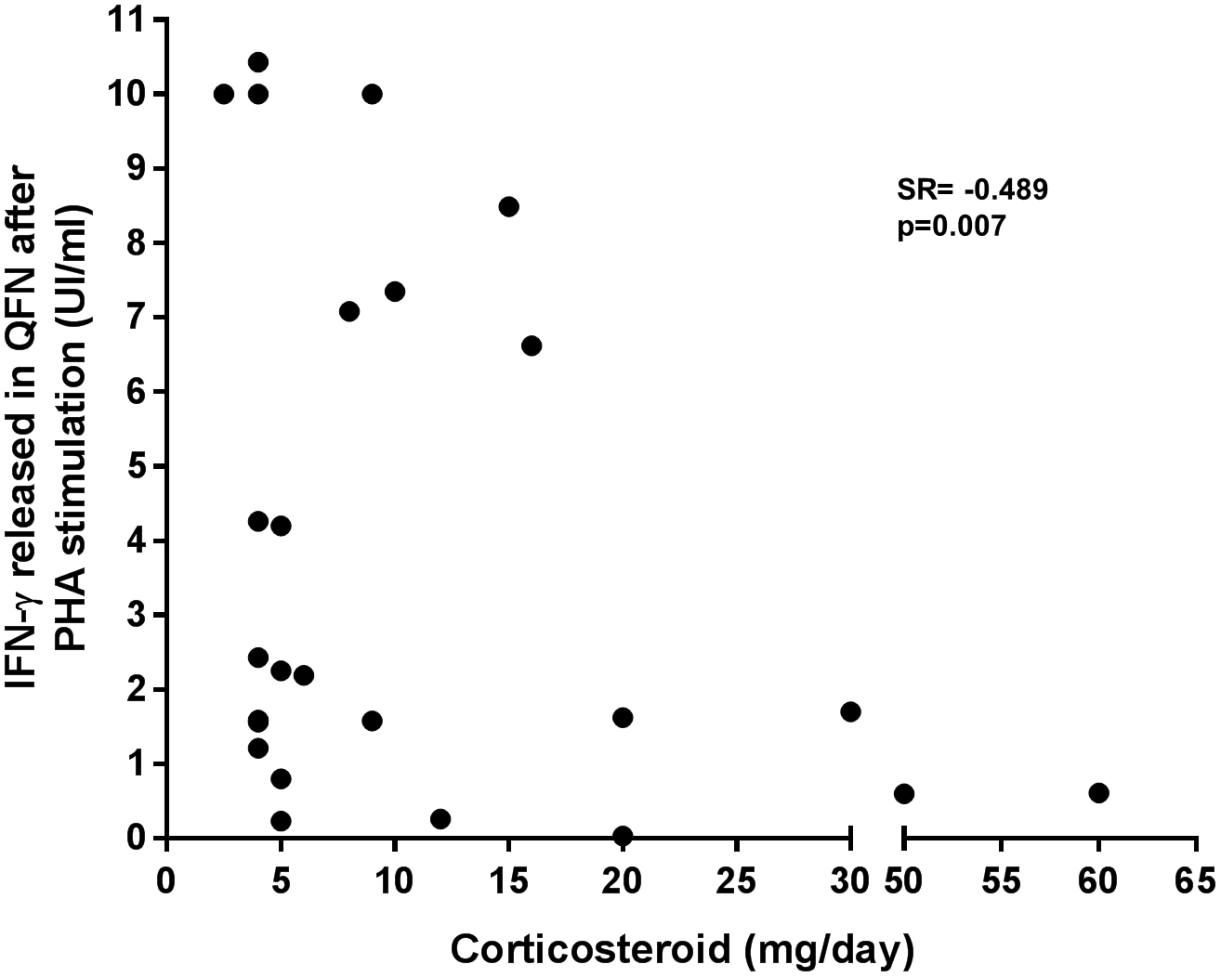
Correlation between the IFN-γ secreted after mitogen (PHA) stimulation and dose (mg/day) of corticosteroid administered. Association between the IFN-γ response on QFN-G-IT and corticosteroid doses was assessed using Spearman correlation coefficient. Measurements >10IU/mL were set as 10IU/mL according to the upper limit of the assay and manufacturer’s instructions. It was not possible to assess the IFN-γ response after PHA stimulation in T-SPOT.TB assay due to saturation in the control well (>250 spot-forming cells). PHA: Phytohaemagglutinin; QFN-G-IT: QuantiFERON-TB Gold In-Tube; SR: Spearman’s Rho.

The SFC and the IFN-γ response in T-SPOT.TB and QFN-G-IT was assessed in those positive responders after specific antigen stimulation, and evaluated regarding the underlying inflammatory disease. No significant differences were found between study groups (p = 0.101 and p = 0.574 respectively, data analysed with Kruskall-Wallis test). In order to further study the impact of different IMIDs on clinical assay accuracy, the amount of IFN-γ released after PHA stimulation in QFN-G-IT was analysed in the overall population. Interestingly, in Crohn’s and rheumatic disease patients, the IFN-γ cytokine secreted by sensitized T cells after PHA stimulation was significantly lower with respect to psoriasis and healthy controls (Fig 2; p<0.0001 for all comparisons). Psoriatic patients were the only ones not receiving corticosteroids. A correlation between the IFN-γ secreted after mitogen stimulation and dose (mg/day) of corticosteroid administered was performed. There was a negative moderate correlation (Fig 3; Spearman’s Rho = -0.489; p = 0.007).

## Discussion

Investigations on IGRAs in high risk individuals are still limited, and it is necessary to compare and study how IMIDs with different immunosuppressive regimen can affect *in vitro* assays’ accuracy. Our research group have previously assessed in two independent studies the usefulness of IGRAs on patients with arthritic [12] and psoriatic [13] diseases, indicating that IGRAs seem to be helpful for LTBI diagnosis in such populations. In both studies we found that positive results for T-SPOT.TB/QFN-G-IT and the amount of T-cell response were not affected by clinical therapeutic profile. However, the effect of therapy on IFN-γ detection may depend on IMID type and its concomitant therapy. Here, we compare three different types of IMIDs (psoriasis, rheumatic inflammatory disease and Crohn’s disease) from patients enrolled in the same study setting. Furthermore, the study shows that accuracy of T-SPOT.TB and QFN-G-IT is differentially affected regarding the IMID type and its concomitant drug-treatment. The overall number of positive results obtained here by TST and IGRAs were reduced in patients suffering Crohn’s disease with respect to those with rheumatic disorders or psoriasis. Similarly, Ramos JM. et al. conducted a study using QFN-G-IT and TST assays in our region, observing that positive results were negatively affected by immunosuppressive therapy in patients with inflammatory bowel diseases. However indeterminate results obtained were low, therefore, no associations were established [14]. Low rates of positive IGRAs results on patients with inflammatory bowel diseases under azathioprine have been previously mentioned by others when they are compared with bowel disease patients under other drug-regimens or without immunosuppressive treatment [15]. Interestingly, the majority of Crohn’s disease patients included in our study were under this immunosuppressant drug.

Generally, the frequency of QFN-G-IT indeterminate results has been linked to patients with chronic inflammatory diseases under glucocorticoid use [8, 16–19]. In line with this, our results reveal that number of IGRA’s indeterminate results is even higher in Crohn’s disease patients under azathioprine. Moreover, the induced-mitogen response was reduced in this group and also in patients with rheumatic diseases with respect to those patients with psoriasis. Intriguingly, the 33.3% of Crohn’s disease patients and the 40% of individuals with rheumatic diseases were on corticosteroid therapy. Bélard E. et al. found a significant negative effect of prednisolone on IFN-γ response, indeterminate QFN-G-IT results and positive TST results. They also compared the IFN-γ response after mitogen stimulation in several therapy groups, and found that it was reduced in patients with corticosteroid treatment when they were compared with those not receiving treatment or other therapy without corticosteroid [16]. In order to study whether the immune response was influenced by corticosteroid treatment, we correlated both IFN-γ released upon PHA stimulation with dose (mg/day) of corticosteroid administered, observing that this response diminished when the dose of corticosteroids augmented. In addition, a recent study has assessed the impact of corticosteroids on QFN-G-IT suggesting that they can significantly impair IGRAs accuracy [20]. Consequently, we have to pay attention when ruling out LTBI in this specific group of patients due to false-negative TST/IGRAs and low IFN-γ cytokine secretion that can lead to indeterminate results. In this direction, some studies report that a long *in vitro* stimulation of T cells (6–9 days) can detect a memory response and may be can be used to identify LTBI individuals who resulted to have negative IGRAs. More studies need to confirm these data, however, this could be a useful tool on patients under immunosuppressive treatment who have high risk of developing TB [21–23].

Taken together, here we describe how IMIDs can negatively affect the accuracy of IGRAs, being Crohn’s disease patients the most affected individuals due to their concomitant immunosuppressant drug-profile and impaired immune response. Therefore, in the absence of a gold standard, it seems prudent to diagnose LTBI using both the TST and IGRAs with the aim of detecting all possible cases in patients with IMIDs [24, 25], giving special attention to those with a poor accuracy of IGRAs and/or TST such as Crohn’s disease. In addition, the use of TST in psoriasis has to be taken with caution. Previous observations indicate that psoriasis can produce a TST hyper-reactivity due to a skin over-reaction to a broad range of antigens. Therefore, it is necessary to re-evaluate a positive TST reaction in these patients because in most situations it could lead to a LTBI over-diagnosis [26].

It is still necessary to make efforts for an accurate LTBI diagnosis in IMIDs’ patients. In this direction, a new improved IGRA version called QFN-Plus has appeared. This assay includes new designed peptides form *M*. *tuberculosis* capable to induce specific CD4 and CD8 T cells. Data about its better accuracy over classical IGRAs is still limited, and there are not yet results evidencing a better sensitivity and specificity in this population [27–30]. Another encouraging approach seems to be the study of other cytokines different from IFN-γ. In the last years, the cytokine called IFN-γ-induced protein-10 (IP-10) has emerged as a promising biomarker for LTBI diagnosis that enables to rise sensitivity in conjunction with IFN-γ [31–34]. We should focus investigations on this direction: improving LTBI diagnostics on high-risk individuals, increasing sensitivity and minimizing false-negative and indeterminate results.

In conclusion, clinical accuracy of IGRAs for LTBI diagnosis seems to be differentially affected by the IMID type. Particularly, Crohn’s disease and/or its concomitant immunosuppressive drug-profile could negatively affect accuracy of T-SPOT.TB and QFN-G-IT when compared with psoriasis orinflammatory rheumatic diseases. Therefore, it is important to be prudent when diagnosing LTBI in this kind of patients due to the high frequency of indeterminate results and an attenuated IFN-γ response.
